# Carbapenem and Multidrug Resistance Among Gram-Negative Bacilli in a County Emergency Clinical Hospital from Western Romania: A Retrospective Study, 2024–2025

**DOI:** 10.3390/medicina62101869

**Published:** 2026-09-27

**Authors:** Lenuta Varvari Timis, Andrei Daescu, Corina Musuroi, Silvia Ioana Musuroi, Adela Voinescu, Andrei Cosnita, Teodora Daniela Marți, Delia Muntean, Monica Licker

**Affiliations:** 1 Doctoral School, “Victor Babes” University of Medicine and Pharmacy, Eftimie Murgu Square No. 2, 300041 Timisoara, Romania; varvari.lenuta@umft.ro (L.V.T.); silvia.musuroi@umft.ro (S.I.M.); adela.voinescu@umft.ro (A.V.); 2 Multidisciplinary Research Center on Antimicrobial Resistance (MULTI-REZ), “Victor Babes” University of Medicine and Pharmacy, Eftimie Murgu Square 2, 300041 Timisoara, Romania; corina.musuroi@umft.ro (C.M.); muntean.delia@umft.ro (D.M.); licker.monica@umft.ro (M.L.); 3 Arad County Emergency Clinical Hospital, Str. Andrényi Károly Nr. 24, 310037 Arad, Romania; marti.teodora@uvvg.ro; 4 Microbiology Laboratory, “Pius Brinzeu” County Clinical Emergency Hospital, 300723 Timisoara, Romania; 5 Discipline of Clinical Skills, Department I Nursing, “Victor Babes” University of Medicine and Pharmacy, 300041 Timisoara, Romania; 6 Surgery & Ophthalmology Department, “Victor Babes” University of Medicine and Pharmacy, 300041 Timisoara, Romania; cosnita.dan@umft.ro; 7 Department of Medicine, Vasile Goldis Western University of Medicine and Pharmacy, 310025 Arad, Romania

**Keywords:** Gram-negative bacilli, antimicrobial resistance, carbapenem resistance, multidrug resistance, intensive care unit, *Escherichia coli*, *Klebsiella pneumoniae*, *Acinetobacter baumannii*, *Pseudomonas aeruginosa*, Romania

## Abstract

*Background and Objectives*: Antimicrobial resistance among Gram-negative bacilli is a major clinical challenge, particularly in hospitals where carbapenem-resistant and multidrug-resistant organisms limit therapeutic options. This study aimed to characterize the distribution and resistance profiles of Gram-negative bacilli isolated in a university-affiliated hospital in western Romania during 2024–2025, focusing on temporal variation, intensive care unit (ICU) origin, carbapenem resistance, and multidrug resistance (MDR). *Materials and Methods*: This retrospective, single-center study included 8931 Gram-negative isolates recovered from clinical specimens between January 2024 and December 2025. Bacterial identification was performed using the VITEK 2 Compact system and/or MALDI-TOF mass spectrometry, whereas antimicrobial susceptibility testing was performed using the VITEK 2 system and interpreted according to EUCAST criteria. Carbapenem resistance was defined as resistance to meropenem, imipenem, or ertapenem for Enterobacterales and to meropenem or imipenem for *Pseudomonas aeruginosa* and *Acinetobacter baumannii*. MDR was defined as resistance to at least one agent in at least three antimicrobial classes. Factors associated with MDR were evaluated using multivariable logistic regression. *Results*: *Escherichia coli* was the most frequent isolate (36.0%), followed by *Klebsiella pneumoniae* (22.8%), *P. aeruginosa* (9.6%), *A. baumannii* (9.4%), and *Proteus mirabilis* (8.0%). Among 8708 isolates evaluable for both outcomes, carbapenem resistance was 30.8% and MDR was 44.5%; both were significantly more frequent in 2025 than in 2024. ICU isolates showed higher carbapenem resistance (56.1% vs. 18.0%) and MDR (65.3% vs. 33.9%) than non-ICU isolates. *A. baumannii* had the highest burden, with 87.4% carbapenem resistance and 87.5% MDR. MDR was independently associated with study year, ICU origin, age group, sex, specimen type, and bacterial species; the strongest association was observed for *A. baumannii* versus *E. coli* (adjusted OR 23.45, 95% CI 18.38–29.91). *Conclusions*: Carbapenem resistance and MDR were frequent, particularly among ICU isolates and high-risk pathogens. The findings support intensified local surveillance, antimicrobial stewardship, and targeted infection prevention and control measures.

## 1. Introduction

Antimicrobial resistance (AMR) has become one of the most important threats to modern healthcare, reducing the effectiveness of commonly used antibiotics and increasing the clinical burden of bacterial infections worldwide. Gram-negative bacteria are of particular concern because of their high clinical relevance and multiple intrinsic and acquired resistance mechanisms. The global spread of resistant Gram-negative pathogens has important consequences for empirical therapy, infection control, hospital costs, and patient outcomes, making continuous surveillance a core component of antimicrobial resistance control strategies [1,2,3].

The COVID-19 pandemic further complicated global efforts to control AMR. Diagnostic uncertainty and concerns regarding bacterial co-infection resulted in extensive empirical antibiotic prescribing, although confirmed bacterial co-infection was considerably less frequent. A rapid review and meta-analysis estimated that 74.6% of patients with COVID-19 received antibiotics, whereas bacterial co-infection was identified in only 8.6%, suggesting substantial potentially unnecessary antibiotic exposure [4]. This selective pressure, together with disruptions to antimicrobial stewardship and infection prevention and control, may have facilitated the emergence and transmission of resistant hospital pathogens. Although pooled evidence did not demonstrate a uniform statistically significant increase in Gram-negative resistance across all settings, a significant increase was observed in settings where enhanced infection-prevention or antimicrobial-stewardship measures were not reported [5]. These observations emphasize the importance of maintaining antimicrobial stewardship, infection-control measures, and AMR surveillance during healthcare crises.

Among Gram-negative bacilli, Enterobacterales and non-fermenting Gram-negative organisms are frequently recovered from urinary, respiratory, bloodstream, wound, and other clinical specimens. Their distribution varies according to patient population, hospital setting, specimen type, and local antimicrobial use patterns. Routine microbiology laboratory data therefore provide valuable information for describing local epidemiology and detecting changes in resistance profiles over time, particularly in hospital settings where empirical treatment decisions may precede full antimicrobial susceptibility results [6,7,8].

*Escherichia coli* and *Klebsiella pneumoniae* are among the most clinically important Enterobacterales, whereas *Acinetobacter baumannii* and *Pseudomonas aeruginosa* represent major non-fermenting pathogens, particularly in intensive care units. Resistance to broad-spectrum cephalosporins, fluoroquinolones, aminoglycosides, and carbapenems in these organisms limits treatment options and contributes substantially to the burden of multidrug-resistant Gram-negative infections [9,10,11,12,13,14,15,16].

Carbapenem resistance and multidrug resistance are key indicators of the severity of antimicrobial resistance among Gram-negative bacilli. Carbapenems are often used as last-line agents for severe infections caused by extended-spectrum beta-lactamase-producing or otherwise resistant organisms; therefore, resistance to this class is associated with restricted therapeutic options and increased reliance on reserve antimicrobials. Standardized definitions of multidrug resistance, together with harmonized antimicrobial susceptibility interpretation systems, are essential for comparing resistance patterns across studies, hospitals, and countries [17,18,19].

In Europe, antimicrobial resistance shows marked geographical variation, with higher resistance levels frequently reported in southern, central, and eastern European countries. Romania has a substantial burden of antimicrobial resistance, particularly among Gram-negative pathogens such as *K. pneumoniae*, *A. baumannii*, *P. aeruginosa*, and resistant Enterobacterales. Local hospital-based studies therefore remain important for guiding empirical therapy, antimicrobial stewardship, and infection prevention strategies. The present study aimed to characterize the distribution of Gram-negative bacilli and their antimicrobial resistance profiles in a university-affiliated hospital in western Romania during 2024–2025, with emphasis on major pathogens, temporal differences, ICU versus non-ICU isolates, carbapenem resistance, and multidrug resistance [20,21,22,23].

## 2. Materials and Methods

### 2.1. Study Design and Setting

This retrospective, single-center observational study was conducted in the Clinical Microbiology Laboratory of the Arad County Emergency Clinical Hospital, a university-affiliated, category II acute-care hospital in western Romania. The hospital has 1315 inpatient beds and provides medical services across 11 locations. Its critical care structure includes two general adult intensive care units with 44 beds in total, comprising 27 beds in ICU I and 17 beds in ICU II. Additional specialized critical care capacity includes six pediatric ICU beds, nine beds dedicated to adult respiratory and infectious diseases, six obstetric–gynecologic ICU beds, two post-anesthesia surveillance beds for otorhinolaryngology and ophthalmology, and 15 neonatal intensive care beds. The general adult ICUs manage postoperative patients and emergency admissions requiring continuous monitoring and treatment for acute respiratory, cardiovascular, renal, or multiple-organ dysfunction. The hospital also covers a broad range of medical and surgical specialties, including internal medicine, cardiology, gastroenterology, hematology, general and specialty surgery, neurology, urology, nephrology, pediatrics, psychiatry, infectious diseases, pneumology, obstetrics and gynecology, neonatology, oncology, otorhinolaryngology, ophthalmology, dermatology, rehabilitation, and palliative care. The study included Gram-negative bacilli isolated from clinical specimens processed between 1 January 2024 and 31 December 2025. The study was designed to characterize the distribution of Gram-negative bacilli and their antimicrobial resistance profiles, with particular emphasis on *E. coli*, *K. pneumoniae*, *A. baumannii*, and *P. aeruginosa*. Temporal differences between 2024 and 2025 and differences between intensive care unit and non-ICU isolates were also evaluated.

### 2.2. Data Source and Study Population

Microbiological and demographic data were retrospectively extracted from the laboratory information system. The variables collected included the date of specimen processing, patient sex, age category, hospital ward, specimen type, bacterial species, and antimicrobial susceptibility results.

All Gram-negative bacilli recovered from clinical specimens during the study period and identified to the species or genus level were eligible for inclusion. Specimens were categorized as urine, respiratory samples, blood cultures, wound and pus specimens, and other or unspecified specimen types. Respiratory samples included sputum, tracheal aspirates, bronchoalveolar lavage fluid, and other lower respiratory tract specimens, as recorded in the laboratory database. Isolates were classified as originating from ICU or non-ICU wards according to the patient’s location at the time of specimen collection.

Adult and pediatric isolates were classified according to an age threshold of 18 years. Records without valid bacterial identification were excluded. For analyses of resistance to individual antibiotics, susceptibility results that were missing or could not be categorized as susceptible (S), susceptible with increased exposure (I), or resistant (R) were excluded from the denominator for the respective antibiotic.

### 2.3. Specimen Collection and Microbiological Processing

Clinical specimens were collected by trained healthcare personnel as part of routine diagnostic care and processed according to the standard operating procedures of the hospital microbiology laboratory. Collection procedures varied according to specimen type and were intended to minimize contamination and preserve microorganism viability during transport.

Urine specimens were collected in sterile containers, predominantly as clean-catch midstream samples or, when clinically indicated, from urinary catheters. Respiratory specimens included sputum, tracheal aspirates, and bronchoalveolar lavage samples. Blood specimens were inoculated into aerobic and anaerobic BACTEC blood culture bottles and processed using the automated BACTEC blood culture system (Becton, Dickinson and Company, Franklin Lakes, NJ, USA) available in the laboratory. Wound specimens and pus samples were collected using sterile swabs or aspiration, depending on the anatomical site and clinical indication.

Swab-based specimens and other samples requiring transport media were collected using COPAN Diagnostics transport devices containing Stuart, Amies, or Cary–Blair medium, selected according to the specimen type and intended microbiological investigation. The collection devices were manufactured by COPAN Italia S.p.A. (Brescia, Italy).

Specimens were inoculated onto culture media appropriate for the sample type and incubated under routine laboratory conditions. Bacterial growth was assessed according to colony morphology, specimen-specific quantitative or semiquantitative criteria, and the presence of predominant or monomorphic growth. Cultures considered contaminated, polymicrobial without a predominant clinically relevant pathogen, or representative of commensal flora were excluded in accordance with routine laboratory criteria.

During the study period, the laboratory workflow also included automated Gram staining, CO_2_ incubation, and automated culture-plate inoculation and labeling systems. Potentially infectious specimens were handled in a Class II biological safety cabinet to reduce occupational exposure to biological agents. These components were progressively introduced as part of an integrated automated laboratory platform whose implementation began in 2023 and was completed in 2025.

### 2.4. Bacterial Identification and Antimicrobial Susceptibility Testing

Gram-negative isolates were identified within the routine automated workflow of the hospital microbiology laboratory. The VITEK 2 Compact system (bioMérieux, Marcy-l’Étoile, France), routinely used in the laboratory since 2016, was employed for automated microbial identification using Gram-negative identification cards and for antimicrobial susceptibility testing. Antimicrobial susceptibility testing was performed using organism-appropriate VITEK 2 AST cards, with minimum inhibitory concentration (MIC) determination according to the manufacturer’s instructions. Identification and AST cards were selected according to the bacterial organism and the routine laboratory workflow.

Beginning in 2023, matrix-assisted laser desorption/ionization time-of-flight mass spectrometry (MALDI-TOF MS) was progressively introduced into the microbial identification workflow as part of an integrated automated laboratory platform, with implementation completed in 2025. The MALDI-TOF MS system was manufactured by Bruker Daltonics GmbH & Co. KG (Bremen, Germany). Depending on system availability and the routine laboratory workflow, bacterial identification was performed using the VITEK 2 Compact system or MALDI-TOF MS, whereas antimicrobial susceptibility testing was performed using the VITEK 2 system.

Antimicrobial susceptibility results were interpreted according to the European Committee on Antimicrobial Susceptibility Testing breakpoint tables applicable to each study year: EUCAST version 14.0 for isolates recovered in 2024 and version 15.0 for isolates recovered in 2025. Results were categorized as susceptible (S), susceptible, increased exposure (I), or resistant (R). For resistance-rate calculations, only isolates categorized as R were considered resistant.

Only antimicrobial susceptibility results recorded in the laboratory information system and categorized as susceptible (S), susceptible, increased exposure (I), or resistant (R) were included in the analyses. Missing results and results that could not be categorized as S, I, or R were excluded from the corresponding denominators. Colistin susceptibility results were included only when obtained using the laboratory’s validated MIC-based testing workflow.

### 2.5. Resistance Definitions and Study Outcomes

The primary outcomes were resistance to individual antibiotics, carbapenem resistance, and multidrug resistance.

Carbapenem resistance was defined as resistance to at least one carbapenem included in the susceptibility panel. For Enterobacterales, carbapenem resistance included resistance to meropenem, imipenem, or ertapenem. For *P. aeruginosa* and *A. baumannii*, it was defined as resistance to meropenem or imipenem.

Multidrug resistance was operationally defined as resistance to at least one antimicrobial agent in at least three antimicrobial classes, based on the valid susceptibility results available in the laboratory database. Isolates were classified as evaluable for carbapenem resistance or MDR only when sufficient valid susceptibility results were available to apply the respective definition.

Extensively drug-resistant (XDR) and pandrug-resistant (PDR) phenotypes were not evaluated because the routine species-specific susceptibility panels did not consistently include all antimicrobial categories required for their reliable classification, and missing or untested results could not be interpreted as either susceptible or resistant.

The “other Gram-negative bacilli” category comprised all Gram-negative organisms not individually presented among the five most frequently isolated species.

### 2.6. Statistical Analysis

The unit of analysis was the microbiological isolate. Categorical variables were summarized as absolute frequencies and percentages. Percentages were calculated using the number of isolates with available data for each variable as the denominator. For antimicrobial susceptibility results, resistance rates were calculated as the number of resistant isolates divided by the number of isolates with valid susceptibility results, using the formula R/(S + I + R). Non-S/I/R results and missing susceptibility data were excluded from the respective denominators.

Comparisons between isolates recovered in 2024 and 2025 were performed using the chi-square test or Fisher’s exact test, as appropriate. These tests were used to compare distributions according to sex, age category, hospital setting, specimen type, bacterial species, and resistance phenotypes. Carbapenem resistance and multidrug resistance were also compared according to year, hospital setting, and bacterial species.

A multivariable logistic regression model was used to identify factors independently associated with MDR. The dependent variable was MDR status. The model included study year, ICU status, age group, sex, specimen type, and bacterial species as independent variables. Results were reported as adjusted odds ratios with 95% confidence intervals. The reference categories were 2024, non-ICU setting, pediatric patients, female sex, urine samples, and *E. coli*. Statistical significance was defined as a two-sided *p*-value < 0.05.

All statistical analyses were performed using R version 4.5.0 (11 April 2025 ucrt, R Foundation for Statistical Computing, Vienna, Austria) within the RStudio version (2025.5.0.496) environment (Posit Software, PBC, Boston, MA, USA).

### 2.7. Ethical Considerations

The study protocol was approved by the Ethics Committee of the Arad County Emergency Clinical Hospital. Written informed consent for the use of anonymized clinical and laboratory data for scientific purposes was obtained through the general consent form signed by patients at hospital admission. The study was conducted in accordance with the principles of the Declaration of Helsinki and applicable national data-protection regulations.

## 3. Results

### 3.1. Baseline Characteristics and Specimen Distribution

A total of 8931 Gram-negative isolates were included, of which 4916 were recorded in 2024 and 4015 in 2025 (Table 1). Most isolates were obtained from female patients (55.8%) and adults (98.0%), while 33.8% originated from intensive care units. Urine was the most frequent specimen type (42.6%), followed by respiratory samples (25.0%), wound and pus specimens (21.3%), and blood cultures (2.1%).

Compared with 2024, the proportion of ICU isolates was lower in 2025 (32.5% vs. 34.9%, *p* = 0.016). Respiratory samples (22.6% vs. 27.0%, *p* < 0.001) and wound and pus specimens (20.1% vs. 22.3%, *p* = 0.014) were also less frequent in 2025. Other or unspecified specimens accounted for 9.0% of isolates and were more frequent in 2025 than in 2024 (13.8% vs. 5.1%, *p* < 0.001). No significant differences were observed for sex, age category, urinary specimens, or blood cultures.

### 3.2. Species Distribution of Gram-Negative Bacilli

The species distribution is presented in Table 2. *E. coli* was the most frequent organism, accounting for 3213 isolates (36.0%), followed by *K. pneumoniae* with 2040 isolates (22.8%), *P. aeruginosa* with 860 isolates (9.6%), *A. baumannii* with 841 isolates (9.4%), and *P.mirabilis* with 715 isolates (8.0%). Other Gram-negative bacilli accounted for 1262 isolates (14.1%).

Between 2024 and 2025, the relative frequency of *K. pneumoniae* decreased from 24.0% to 21.4% (*p* = 0.004), whereas *P. aeruginosa* increased from 9.0% to 10.4% (*p* = 0.020) and *P. mirabilis* from 6.7% to 9.6% (*p* < 0.001). The frequency of other Gram-negative bacilli decreased from 15.0% to 13.0% (*p* = 0.007). No significant differences were observed for *E. coli* or *A. baumannii*.

### 3.3. Antimicrobial Resistance by Antibiotic Class

The overall antimicrobial resistance profile of Gram-negative bacilli is presented in Table 3. The highest resistance rates were observed for ampicillin/sulbactam (54.5%), tigecycline (50.3%), ciprofloxacin (44.5%), trimethoprim/sulfamethoxazole (44.4%), amoxicillin/clavulanic acid (43.1%), and levofloxacin (43.0%).

Resistance to broad-spectrum cephalosporins ranged from 25.8% for cefepime to 35.4% for ceftriaxone, while resistance to individual carbapenems ranged from 21.5% for ertapenem to 27.4% for imipenem. Among aminoglycosides, resistance was lower for amikacin (21.2%) than for gentamicin (33.1%) and tobramycin (33.2%). Resistance rates were 22.0% for colistin and 15.9% for nitrofurantoin. The fosfomycin estimate of 26.5% was based on a comparatively small number of tested isolates (n = 196).

### 3.4. Species-Specific Resistance Profiles of Major Gram-Negative Pathogens

Species-specific resistance profiles are presented in Table 4 and Figure 1. *E. coli* showed low resistance to carbapenems, with rates of 0.7% for meropenem, 1.8% for imipenem, and 1.1% for ertapenem. Resistance was also low for amikacin (2.1%), but higher for ciprofloxacin (28.5%) and trimethoprim/sulfamethoxazole (32.8%).

*K. pneumoniae* showed substantially higher resistance, including 59.0% to ceftriaxone, 52.9% to cefepime, 56.8% to ciprofloxacin, and between 39.0% and 50.4% to individual carbapenems. *A. baumannii* had the highest resistance rates, including 88.5% to meropenem, 88.3% to imipenem, 90.8% to ciprofloxacin, and 81.1% to gentamicin, while colistin resistance was 4.1%. *P. aeruginosa* showed intermediate resistance rates, including 35.9% to ceftazidime, 34.2% to cefepime, 40.3% to piperacillin/tazobactam, 27.0% to meropenem, and 33.7% to imipenem. Selected clinically relevant antibiotics are presented according to organism and availability of valid susceptibility results.

### 3.5. Carbapenem Resistance and Multidrug Resistance by Year, Care Setting, and Species

Among the 8708 isolates evaluable for both outcomes, 2686 (30.8%) were carbapenem-resistant, and 3876 (44.5%) met the MDR definition (Table 5). Both carbapenem resistance and MDR were more frequent in 2025 than in 2024, increasing from 29.0% to 33.2% and from 42.2% to 47.4%, respectively (both *p* < 0.001).

ICU isolates had higher rates of carbapenem resistance (56.1% vs. 18.0%) and MDR (65.3% vs. 33.9%) than non-ICU isolates (both *p* < 0.001). Among the major pathogens, *A. baumannii* had the highest rates of carbapenem resistance and MDR (87.4% and 87.5%), followed by *K. pneumoniae* (50.1% and 58.8%). The corresponding rates were 33.4% and 31.7% for *P. aeruginosa* and 2.6% and 19.6% for *E. coli* (both overall comparisons *p* < 0.001).

### 3.6. Factors Associated with Multidrug Resistance

In the multivariable logistic regression model, isolates recovered in 2025 had higher odds of MDR than those recovered in 2024 (adjusted OR 1.32, 95% CI 1.20–1.46, *p* < 0.001) (Table 6). MDR was also independently associated with ICU origin (adjusted OR 3.18, 95% CI 2.78–3.63), adult age (adjusted OR 2.43, 95% CI 1.58–3.76), and male sex (adjusted OR 1.27, 95% CI 1.15–1.40).

Compared with urine isolates, blood culture isolates had higher odds of MDR (adjusted OR 1.53, 95% CI 1.04–2.24), whereas respiratory and other/unknown specimens had lower odds. Wound and pus specimens were not significantly associated with MDR. Compared with *E. coli*, the odds of MDR were higher for *K. pneumoniae* (adjusted OR 5.09), *A. baumannii* (adjusted OR 23.45), *P. aeruginosa* (adjusted OR 1.66), and other Gram-negative bacilli (adjusted OR 5.21); all significant associations had *p* < 0.001.

## 4. Discussion

This retrospective isolate-based study provides an overview of the distribution and antimicrobial resistance patterns of Gram-negative bacilli recovered in a university-affiliated hospital in western Romania during 2024–2025. *E. coli* and *K. pneumoniae* together accounted for more than half of all isolates, while urine was the most frequent specimen type. A recent study of positive urine cultures from another tertiary hospital in western Romania similarly identified *E. coli* as the predominant pathogen in both inpatients and outpatients, with *Klebsiella* spp. also among the leading isolates. The importance of these two species was also demonstrated in a Romanian study of Gram-negative sepsis, in which 415 cases were caused by *E. coli* and 278 by *K. pneumoniae*. Furthermore, a large study from southern Italy recovered *K. pneumoniae* from urine, blood, respiratory, wound, and other clinical specimens, illustrating its broad clinical distribution. Although direct comparison is limited by differences in study populations and specimen selection, these findings consistently demonstrate the central role of *E. coli* and *K. pneumoniae* in both routine and invasive Gram-negative infections [24,25,26].

The overall resistance profile showed marked species-specific heterogeneity. While *E. coli* retained low resistance to carbapenems and amikacin, *K. pneumoniae* showed high resistance across cephalosporins, fluoroquinolones, aminoglycosides, piperacillin/tazobactam, and carbapenems. In our cohort, 50.1% of evaluable *K. pneumoniae* isolates were carbapenem-resistant and 58.8% met the MDR definition, identifying this species as one of the principal contributors to the local resistance burden. These findings are consistent with previous Romanian evidence documenting the circulation of carbapenemase-producing *K. pneumoniae*. A multicenter Romanian survey identified carbapenemase production in 65 of 75 carbapenem-non-susceptible isolates, predominantly associated with oxacillinase-48-like (OXA-48-like) enzymes, but also with New Delhi metallo-β-lactamase (NDM), *K. pneumoniae* carbapenemase (KPC), and Verona integron-encoded metallo-β-lactamase (VIM). Genomic characterization of carbapenemase-producing isolates from two Romanian hospitals subsequently demonstrated multidrug resistance, colistin resistance or heteroresistance, and the presence of internationally disseminated high-risk lineages, including ST101, ST147, ST258, and ST307. At the European level, the spread of carbapenem-resistant *K. pneumoniae* has similarly been linked to high-risk clones and mobile resistance determinants, with OXA-48-producing ST101 specifically documented in Romania. Although molecular mechanisms were not investigated in the present study, the high phenotypic resistance rates observed locally are compatible with this broader pattern of dissemination [27,28,29].

*A. baumannii* had the highest resistance burden in the present study, with carbapenem resistance and MDR affecting 87.4% and 87.5% of evaluable isolates, respectively. These values exceed the population-weighted European mean carbapenem non-susceptibility of 35.6% reported for invasive *Acinetobacter* spp. isolates between 2013 and 2017, although substantially higher proportions were observed in southern and eastern Europe, at 75.5% and 71.5%, respectively [30]. The geographical contrast is particularly evident when compared with German surveillance data, where the mean carbapenem resistance rate among clinical *A. baumannii* complex isolates was only 4.2% during 2014–2018 [31]. Differences in surveillance periods, specimen composition, patient populations, species identification, and local epidemiology limit direct comparison; nevertheless, our results place the local resistance burden at the upper end of that reported in European surveillance. The low colistin resistance rate observed in our cohort may indicate preserved in vitro activity, but interpretation remains cautious because colistin susceptibility testing is technically demanding and therapeutic use is restricted by toxicity and pharmacokinetic limitations. The observed profile is consistent with the recognized role of MDR *A. baumannii* as a major ICU pathogen and supports intensified infection prevention, rapid detection, and antimicrobial stewardship measures [32].

*P. aeruginosa* showed an intermediate but clinically relevant resistance profile, with carbapenem resistance in 33.4% and MDR in 31.7% of evaluable isolates. In the multicenter European ASPIRE-ICU study, resistance exceeded 20% for all evaluated agents except colistin, while 24.9% of isolates were extensively drug-resistant and 13.2% met the difficult-to-treat resistance definition [33]. A more recent comparison between ICU and non-ICU isolates reported approximately 48–50% resistance to imipenem, 36% resistance to piperacillin/tazobactam among ICU isolates, 16% resistance to amikacin, and no colistin resistance [34]. Compared with that study, our isolates showed lower imipenem resistance (33.7%), a similar amikacin resistance rate (19.6%), slightly higher piperacillin/tazobactam resistance (40.3%), and higher colistin resistance (7.1%). Previous genomic investigation of resistant *P. aeruginosa* in Estonian hospitals also demonstrated the coexistence of carbapenem resistance and MDR and the potential contribution of hospital dissemination of resistant strains [35]. These differences emphasize the marked geographical and hospital-level variability of *P. aeruginosa* resistance and reinforce the need for local, species-specific, and ward-specific antibiograms.

Recent EARS-Net data further illustrate the substantial between-country variation in carbapenem resistance among invasive isolates. In 2024, carbapenem resistance in *K. pneumoniae* was 50.3% in Romania and 60.2% in Greece, compared with 24.0% in Italy and 1.2% in Germany. For *P. aeruginosa*, the corresponding proportions were 34.1% in Romania, 53.4% in Greece, 13.8% in Italy, and 13.1% in Germany. Carbapenem resistance among *Acinetobacter* spp. was also markedly higher in Romania (92.7%), Greece (94.1%), and Italy (74.3%) than in Germany (2.2%). Although EARS-Net data are based on invasive isolates and differences in national coverage and representativeness limit direct comparisons with our mixed-specimen hospital cohort, these findings demonstrate the pronounced geographical heterogeneity of carbapenem resistance across Europe [3].

The multivariable analysis showed that MDR was independently associated with study year, ICU origin, adult age, male sex, specimen type, and bacterial species. ICU origin was one of the strongest non-species-related determinants, with more than threefold higher adjusted odds of MDR compared with non-ICU isolates. Previous studies have similarly identified intensive care exposure and healthcare-related factors as important components of the MDR burden. In a study of MDR Gram-negative bacterial carriage at ICU admission, 6.2% of admissions were associated with carriage, with previous MDR carriage, digestive surgery, liver cirrhosis, and longer previous hospitalization identified as independent risk factors [36]. Among ICU patients with Gram-negative infections, combination antibiotic treatment, healthcare-associated infection, and mechanical ventilation for at least seven days were independently associated with carbapenem-resistant infection [37]. In a community-hospital study, MDR affected 30.8% of Gram-negative isolates, while extended-spectrum beta lactamase (ESBL) production, urinary tract infection, and previous steroid use were independently associated with MDR infection [38]. Although these studies evaluated patient-level exposures that were unavailable in our database, their findings support the association between resistance and prior healthcare exposure, invasive care, antimicrobial pressure, and specific bacterial phenotypes. Importantly, the higher adjusted odds of MDR in 2025 persisted after accounting for ICU origin, specimen type, and species, suggesting that the temporal increase was not attributable solely to changes in isolate composition.

These findings have direct implications for antimicrobial stewardship and infection prevention and control. Empirical treatment protocols should be informed by regularly updated local antibiograms stratified by species, specimen type, and care setting, particularly in ICUs, where the resistance burden was highest. Previous work has shown the practical value of incorporating local microbial ecology and recent surveillance cultures into empirical treatment algorithms for hospital-acquired pneumonia, with the aim of maintaining appropriate antimicrobial coverage while limiting unnecessary broad-spectrum therapy [39]. Current infection prevention guidance also emphasizes risk-based screening, isolation precautions, source and contact investigation, and coordinated organizational measures for controlling multidrug-resistant organisms [40]. In addition, surveillance approaches that account for patient movement between hospitals may provide more robust estimates of antimicrobial resistance dissemination than isolated institutional reporting.

Beyond antimicrobial stewardship and infection-prevention measures, the high burden of MDR observed in this study highlights the need to investigate complementary therapeutic strategies. Bacteriophage therapy represents a promising approach because lytic phages can selectively target resistant bacteria and may act synergistically with conventional antibiotics. Advanced delivery systems, including liposomes, hydrogels, and polymeric carriers, are being developed to improve phage stability, bioavailability, and targeted delivery to the site of infection, although standardization, pharmacokinetic characterization, and clinical validation remain necessary. Another emerging approach is the siderophore-mediated “Trojan horse” strategy, in which antibiotics are conjugated to metal-chelating molecules to exploit bacterial metal-uptake pathways and facilitate drug entry across the outer membrane of Gram-negative bacteria. Metallophores themselves also represent potential therapeutic targets because they enable the acquisition of essential metals and contribute to bacterial survival and virulence. Consequently, inhibiting metallophore biosynthesis, secretion, uptake, or receptor interactions may attenuate bacterial pathogenicity and support the development of new antimicrobial classes. Although these strategies remain largely investigational, they may complement surveillance, antimicrobial stewardship, and infection-control interventions in addressing infections caused by MDR Gram-negative pathogens [41].

This study has several limitations. First, its retrospective, single-center design limits the generalizability of the findings to other hospitals and geographical regions. Second, the analysis was isolate-based; therefore, the results describe isolate distributions and resistance patterns and should not be interpreted as patient-level incidence estimates. Third, the laboratory database did not include information on antimicrobial consumption, clinical outcomes, or individual healthcare exposures, preventing the evaluation of their potential relationships with antimicrobial resistance. Molecular resistance mechanisms and carbapenemase types were also not characterized. Finally, antimicrobial susceptibility results were not uniformly available for every organism–antibiotic combination, and the testing panels varied according to bacterial species and routine laboratory workflow. Consequently, the denominators differed between antibiotics, and direct comparisons between resistance percentages should be interpreted cautiously. Consequently, the available data did not permit the reliable classification of XDR or PDR phenotypes. Nevertheless, the large number of isolates, inclusion of multiple specimen types, comparison between ICU and non-ICU settings, and adjusted analysis of MDR determinants provide relevant local evidence for guiding antimicrobial stewardship, surveillance, and infection prevention interventions [42].

To our knowledge, this is the first comprehensive hospital-wide study conducted at the Arad County Emergency Clinical Hospital to integrate the distribution of Gram-negative bacilli across multiple clinical specimen types with resistance profiles for individual antibiotics, carbapenem resistance, multidrug resistance, temporal trends, ICU versus non-ICU comparisons, and a multivariable analysis of factors associated with MDR.

## 5. Conclusions

This study provides a comprehensive local assessment of Gram-negative bacilli and their antimicrobial resistance patterns in a university-affiliated hospital in western Romania, integrating species distribution, resistance to individual antibiotics, carbapenem resistance, MDR, temporal variation, care setting, and independently associated factors. The findings identify ICU isolates and high-risk pathogens, particularly *A. baumannii* and *K. pneumoniae*, as the main contributors to the local burden of carbapenem resistance and MDR, while also indicating an increase in resistance during 2025 that warrants continued monitoring.

These results support the development of targeted infection prevention and control and antimicrobial stewardship strategies. Priority measures should include intensified surveillance in ICUs and other high-risk wards, rapid detection and communication of carbapenem-resistant and MDR isolates, reinforcement of hand hygiene and contact precautions where indicated, environmental cleaning, prevention of device-associated infections, and regular preparation of species- and ward-specific antibiograms. Continued local surveillance will be essential to determine whether the observed resistance trends persist and to evaluate the impact of future infection prevention and antimicrobial stewardship interventions.

## Figures and Tables

**Figure 1:**
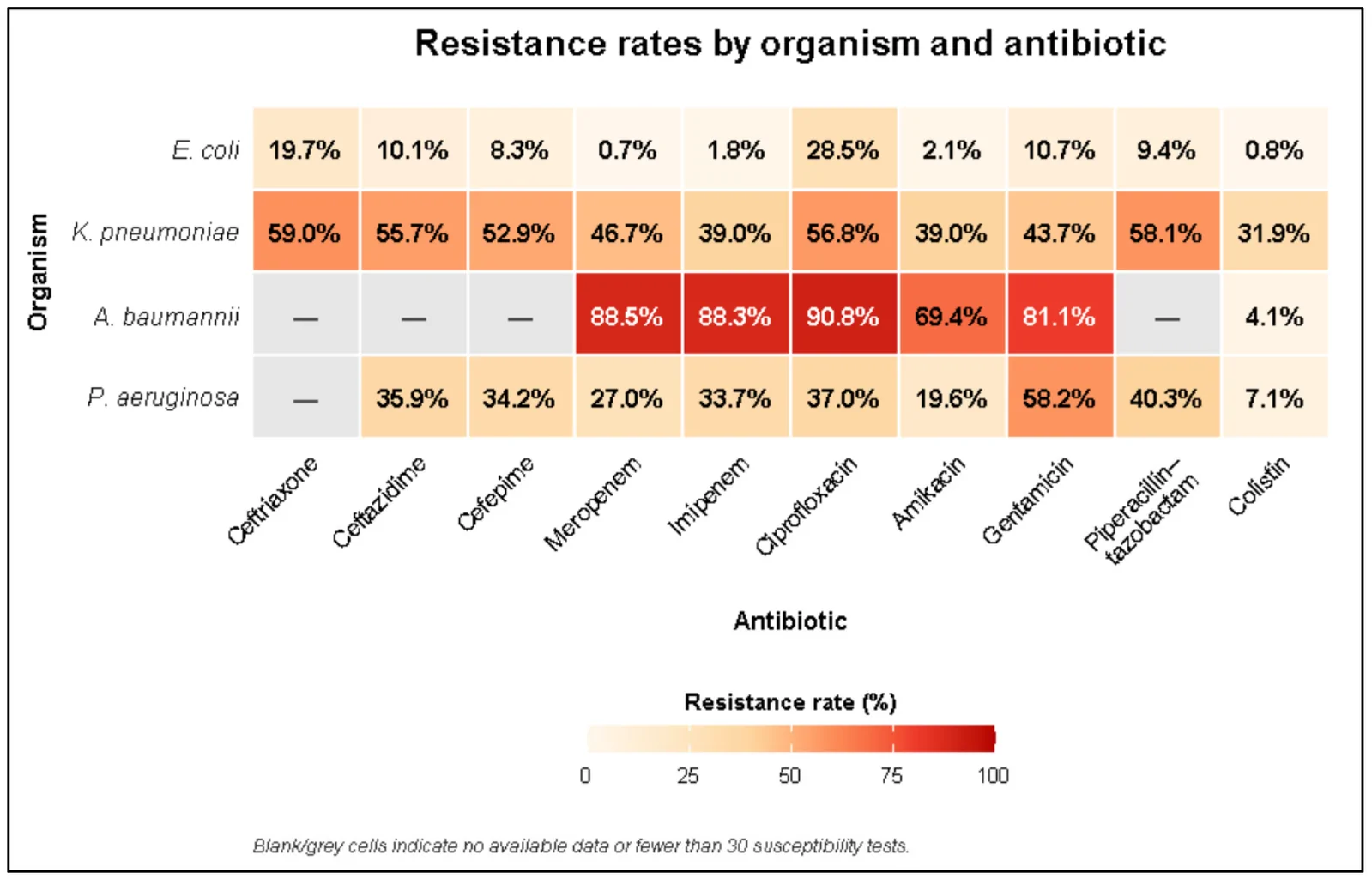
Antimicrobial resistance profiles of major Gram-negative pathogens across selected antibiotics. The heatmap shows the percentage of resistant isolates for selected clinically relevant antibiotics among the four main Gram-negative pathogens: *E. coli*, *K. pneumoniae*, *A. baumannii*, and *P. aeruginosa*. Resistance rates were calculated as the proportion of resistant isolates among all isolates with valid susceptibility results R/(S+I+R). Darker shades indicate higher resistance rates. Only organism–antibiotic combinations with at least 30 valid susceptibility tests were included in the figure. Blank/grey cells indicate that no applicable clinical breakpoint was available or that fewer than 30 valid susceptibility results were recorded.

**Table 1:** Baseline characteristics and specimen sources of Gram-negative isolates, 2024–2025.

Characteristic	Total	2024	2025	*p*-Value
Number of isolates	8931	4916	4015	—
Male sex	3944 (44.2%)	2195 (44.7%)	1749 (43.6%)	0.295
Female sex	4985 (55.8%)	2719 (55.3%)	2266 (56.4%)	0.295
Adult	8750 (98.0%)	4804 (97.7%)	3946 (98.3%)	0.058
Pediatric	179 (2.0%)	111 (2.3%)	68 (1.7%)	0.058
ICU	3018 (33.8%)	1715 (34.9%)	1303 (32.5%)	**0.016**
Non-ICU	5913 (66.2%)	3201 (65.1%)	2712 (67.5%)	**0.016**
Urine	3802 (42.6%)	2138 (43.5%)	1664 (41.4%)	0.052
Respiratory samples	2232 (25.0%)	1326 (27.0%)	906 (22.6%)	**<0.001**
Blood cultures	188 (2.1%)	107 (2.2%)	81 (2.0%)	0.602
Wound/pus specimens	1903 (21.3%)	1095 (22.3%)	808 (20.1%)	**0.014**
Other/unspecified specimens	806 (9.0%)	250 (5.1%)	556 (13.8%)	**<0.001**

Note: Percentages are calculated within each column. For sex and age category, percentages are calculated among isolates with available data. *p*-values compare 2024 vs. 2025 using the chi-square test or Fisher’s exact test, as appropriate. Statistically significant *p*-values (*p* < 0.05) are shown in bold.

**Table 2:** Species distribution of Gram-negative bacilli by study year.

Organism	Total n (%)	2024 n (%)	2025 n (%)	*p*-Value
*E. coli*	3213 (36.0%)	1776 (36.1%)	1437 (35.8%)	0.742
*K. pneumoniae*	2040 (22.8%)	1180 (24.0%)	860 (21.4%)	**0.004**
*A.baumannii*	841 (9.4%)	452 (9.2%)	389 (9.7%)	0.426
*P. aeruginosa*	860 (9.6%)	441 (9.0%)	419 (10.4%)	**0.020**
*P. mirabilis*	715 (8.0%)	328 (6.7%)	387 (9.6%)	**<0.001**
Others	1262 (14.1%)	739 (15.0%)	523 (13.0%)	**0.007**

Note: Percentages are calculated among Gram-negative isolates. *p*-values compare the relative frequency of each organism in 2024 vs. 2025. Statistically significant *p*-values (*p* < 0.05) are shown in bold.

**Table 3:** Antimicrobial resistance rates among Gram-negative bacilli by antibiotic class.

Antibiotic	Class	n Tested	n Resistant	% Resistant
Piperacillin/tazobactam	Beta-lactam/beta-lactamase inhibitor	7293	2164	29.7
Amoxicillin/clavulanic acid	Beta-lactam/beta-lactamase inhibitor	6899	2973	43.1
Ampicillin/sulbactam	Beta-lactam/beta-lactamase inhibitor	2032	1107	54.5
Ceftriaxone	Broad-spectrum cephalosporins	6393	2265	35.4
Cefotaxime	Broad-spectrum cephalosporins	6473	2248	34.7
Ceftazidime	Broad-spectrum cephalosporins	7416	2200	29.7
Cefepime	Broad-spectrum cephalosporins	7231	1868	25.8
Meropenem	Carbapenems	7779	1984	25.5
Imipenem	Carbapenems	8409	2304	27.4
Ertapenem	Carbapenems	6436	1381	21.5
Ciprofloxacin	Fluoroquinolones	8263	3675	44.5
Levofloxacin	Fluoroquinolones	7331	3154	43.0
Amikacin	Aminoglycosides	8563	1818	21.2
Gentamicin	Aminoglycosides	8026	2655	33.1
Tobramycin	Aminoglycosides	7907	2627	33.2
Trimethoprim/sulfamethoxazole	Folate pathway inhibitors	7412	3291	44.4
Colistin	Polymyxins	7799	1715	22.0
Tigecycline	Glycylcyclines/tetracyclines	3237	1628	50.3
Fosfomycin	Other	196	52	26.5
Nitrofurantoin	Other	2576	409	15.9

Note: Resistance percentages were calculated as the number of resistant isolates divided by the number of isolates with valid susceptibility results, using the formula R/(S + I + R). Non-S/I/R results were excluded from the denominator.

**Table 4:** Selected species-specific antimicrobial resistance profiles among major Gram-negative pathogens.

Organism	Antibiotic	n Tested	n Resistant	% Resistant
*E. coli*	Ceftriaxone	2885	567	19.7
	Cefepime	2932	242	8.3
	Ciprofloxacin	2971	847	28.5
	Amikacin	3156	65	2.1
	Gentamicin	3158	339	10.7
	Piperacillin/tazobactam	2880	272	9.4
	Meropenem	2802	19	0.7
	Imipenem	3014	54	1.8
	Ertapenem	2857	32	1.1
	Trimethoprim/sulfamethoxazole	2909	954	32.8
	Nitrofurantoin	2055	55	2.7
*K. pneumoniae*	Ceftriaxone	1858	1097	59.0
	Cefepime	1872	990	52.9
	Ciprofloxacin	1965	1117	56.8
	Amikacin	2016	787	39.0
	Gentamicin	2026	886	43.7
	Piperacillin/tazobactam	1947	1131	58.1
	Meropenem	1824	852	46.7
	Imipenem	1983	773	39.0
	Ertapenem	1917	966	50.4
	Colistin	1857	593	31.9
	Tigecycline	1108	696	62.8
	Trimethoprim/sulfamethoxazole	1920	990	51.6
*A. baumannii*	Ciprofloxacin	775	704	90.8
	Levofloxacin	698	642	92.0
	Amikacin	785	545	69.4
	Gentamicin	837	679	81.1
	Tobramycin	769	533	69.3
	Meropenem	710	628	88.5
	Imipenem	823	727	88.3
	Colistin	731	30	4.1
	Tigecycline	47	31	66.0
	Trimethoprim/sulfamethoxazole	772	623	80.7
*P. aeruginosa*	Ceftazidime	775	278	35.9
	Cefepime	757	259	34.2
	Ciprofloxacin	797	295	37.0
	Gentamicin	165	96	58.2
	Tobramycin	776	207	26.7
	Piperacillin/tazobactam	784	316	40.3
	Meropenem	768	207	27.0
	Imipenem	781	263	33.7
	Colistin	762	54	7.1

**Table 5:** Carbapenem resistance and multidrug resistance by year, care setting, and bacterial species.

Characteristic	Category	n	Carbapenem Resistance n (%)	MDR n (%)	*p*-Value for Both Outcomes
Overall	Total	8708	2686 (30.8%)	3876 (44.5%)	
Year	2024	4853	1408 (29.0%)	2048 (42.2%)	**<0.001**
	2025	3855	1278 (33.2%)	1828 (47.4%)	
Hospital setting	ICU	2936	1647 (56.1%)	1917 (65.3%)	**<0.001**
	Non-ICU	5772	1039 (18.0%)	1959 (33.9%)	
Microorganism	*E. coli*	3136	82 (2.6%)	614 (19.6%)	**<0.001**
	*K. pneumoniae*	2033	1018 (50.1%)	1195 (58.8%)	
	*A. baumannii*	833	728 (87.4%)	729 (87.5%)	
	*P. aeruginosa*	830	277 (33.4%)	263 (31.7%)	

Note: Percentages were calculated among isolates evaluable for both carbapenem resistance and MDR. Carbapenem resistance was defined as resistance to meropenem, imipenem, or ertapenem for Enterobacterales, and resistance to meropenem or imipenem for *P. aeruginosa* and *A. baumannii*. For year, hospital setting, and bacterial species, both carbapenem resistance and MDR differed significantly between categories (all *p* < 0.001), using the chi-square test or Fisher’s exact test, as appropriate. Statistically significant *p*-values (*p* < 0.05) are shown in bold.

**Table 6:** Multivariable logistic regression analysis of factors associated with multidrug resistance.

Predictor	Adjusted OR	95% CI	*p*-Value
2025 vs. 2024	1.32	1.20–1.46	**<0.001**
ICU vs. Non-ICU	3.18	2.78–3.63	**<0.001**
Adult vs. Pediatric	2.43	1.58–3.76	**<0.001**
Male vs. Female	1.27	1.15–1.40	**<0.001**
Respiratory samples vs. Urine	0.42	0.35–0.49	**<0.001**
Blood culture vs. Urine	1.53	1.04–2.24	**0.030**
Wound/pus specimens vs. Urine	0.97	0.85–1.11	0.635
Other/unknown vs. Urine	0.56	0.46–0.68	**<0.001**
*K. pneumoniae* vs. *E. coli*	5.09	4.45–5.82	**<0.001**
*A. baumannii* vs. *E. coli*	23.45	18.38–29.91	**<0.001**
*P. aeruginosa* vs. *E. coli*	1.66	1.37–2.00	**<0.001**
Other Gram-negative bacilli vs. *E. coli*	5.21	4.54–5.98	**<0.001**

Note: Logistic regression model adjusted for year, ICU status, age group, sex, specimen type, and bacterial species. Reference categories were 2024, non-ICU, pediatric patients, female sex, urine samples, and *E. coli*. OR, odds ratio; CI, confidence interval; MDR, multidrug resistance. Statistically significant *p*-values (*p* < 0.05) are shown in bold.

## Data Availability

The data supporting the findings of this study are available from the corresponding author upon reasonable request.

## Abbreviations

The following abbreviations are used in this manuscript:

AST	Antimicrobial susceptibility testing
CI	Confidence interval
EUCAST	European Committee on Antimicrobial Susceptibility Testing
I	Susceptible, increased exposure
ICU	Intensive care unit
MDR	Multidrug resistance
MIC	Minimum inhibitory concentration
MALDI-TOF MS	Matrix-assisted laser desorption/ionization time-of-flight mass spectrometry
OR	Odds ratio
R	Resistant
S	Susceptible
n	Number of isolates
spp.	Multiple species within a genus
*A. baumannii*	*Acinetobacter baumannii*
*E. coli*	*Escherichia coli*
*K. pneumoniae*	*Klebsiella pneumoniae*
*P. aeruginosa*	*Pseudomonas aeruginosa*
*P. mirabilis*	*Proteus mirabilis*
AI	Artificial intelligence
AMR	Antimicrobial resistance
ASPIRE-ICU	Advanced understanding of *Staphylococcus aureus* and *Pseudomonas aeruginosa* Infections in EuRopE–Intensive Care Units
COVID-19	Coronavirus disease 2019
ESBL	Extended-spectrum beta-lactamase
KPC	*Klebsiella pneumoniae* carbapenemase
NDM	New Delhi metallo-beta-lactamase
OXA-48	Oxacillinase-48-like carbapenemase
ST	Sequence type
VIM	Verona integron-encoded metallo-beta-lactamase
XDR	Extensively drug-resistant
PDR	Pandrug-resistant
